# Supplementary material for: Complex‐centric proteome profiling by SEC‐SWATH‐MS
Source: Mol Syst Biol. 2019 Jan 14;15(1):e8438. doi: 10.15252/msb.20188438 (PMC6346213; doi:10.15252/msb.20188438)
Supplement: Supplementary file 8 — Dataset EV7 [file MSB-15-e8438-s008.zip › feature_plots_string/Q9UBN7.pdf]

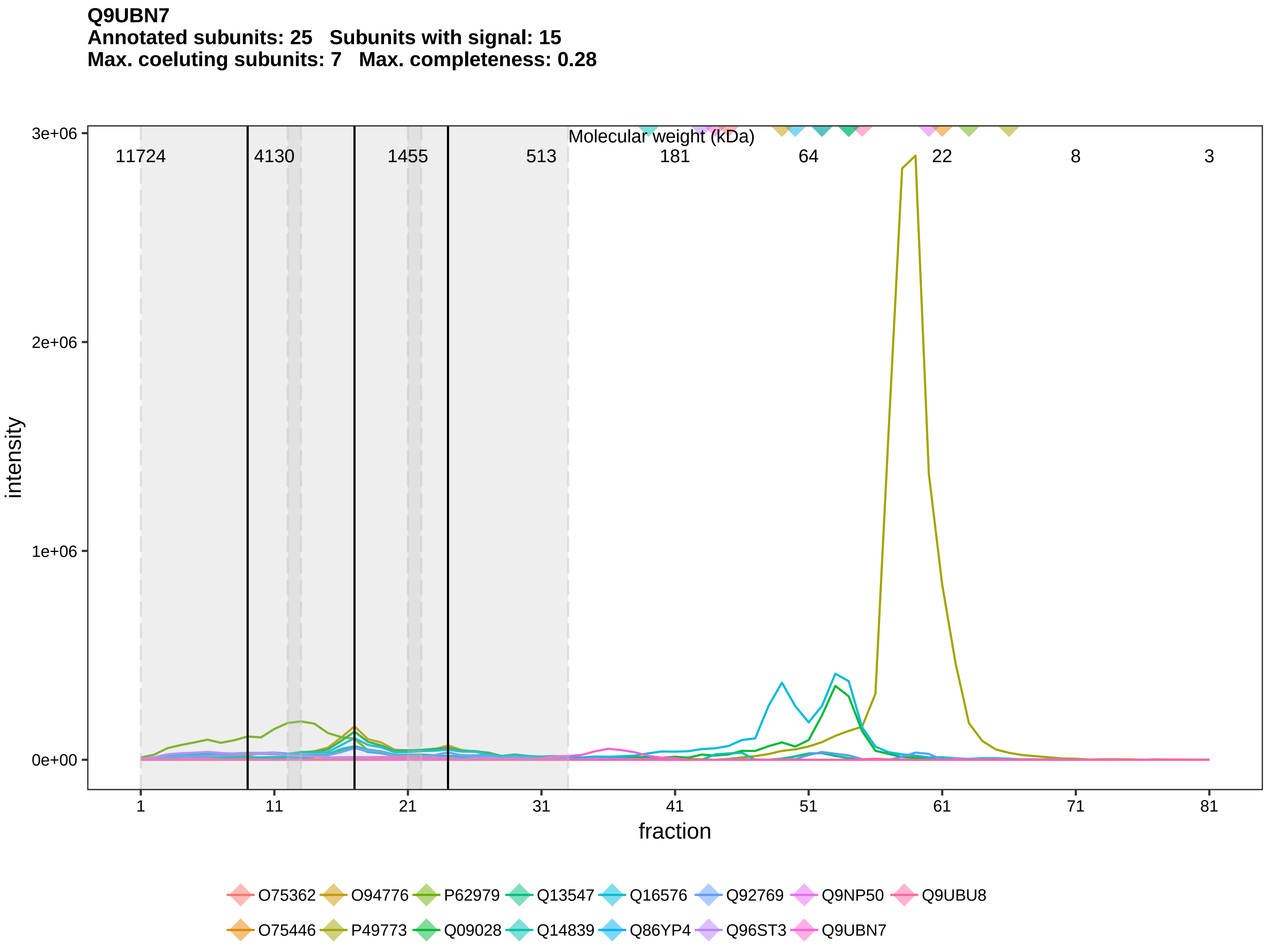
Q9UBN7
Annotated subunits: 25   Subunits with signal: 15
Max. coeluting subunits: 7   Max. completeness: 0.28
Molecular weight (kDa)
11724
4130
1455
513
181
64
22
8
3
intensity
3e+06
2e+06
1e+06
0e+00
fraction
1
11
21
31
41
51
61
71
81
O75362
O94776
P62979
Q13547
Q16576
Q92769
Q9NP50
Q9UBU8
O75446
P49773
Q09028
Q14839
Q86YP4
Q96ST3
Q9UBN7
